# Supplementary material for: Lactate Reprograms Energy and Lipid Metabolism in Glucose-Deprived Oxidative Glioma Stem Cells
Source: Metabolites. 2021 May 18;11(5):325. doi: 10.3390/metabo11050325 (PMC8158503; doi:10.3390/metabo11050325)
Supplement: Supplementary file 1 [file metabolites-11-00325-s001.zip › Figure S1-Effect of lactate on sphere formation and oxygen consumption in glucose-deprived GSCs and re-lated methods..pdf]

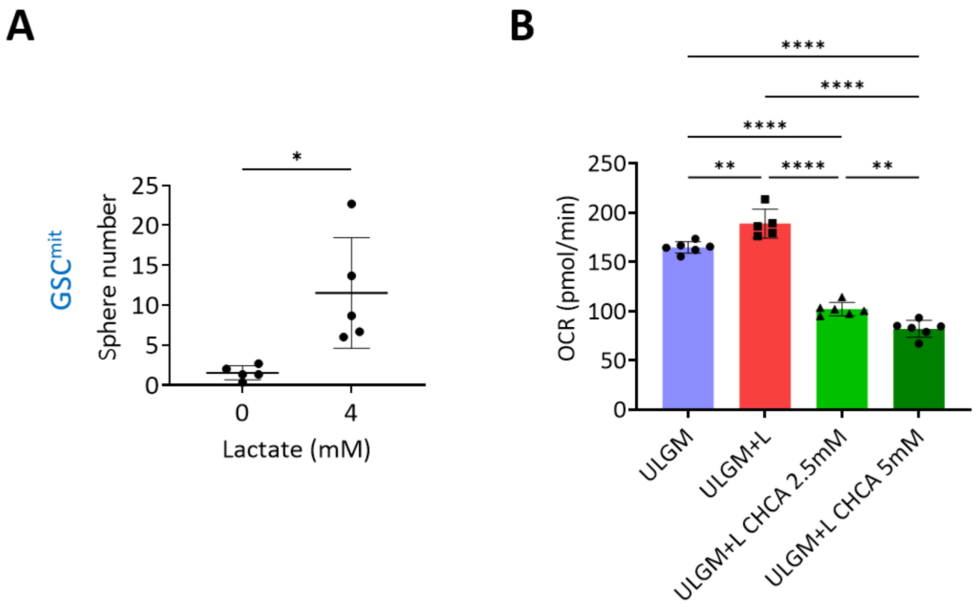

Figure S1. (A) Number of spheres formed by GSC^{mit} in ULGM with or without 4 mM lactate. Data represent the average number of spheres/field for 2 × 10^4 cells plated. Means ± SD are from n= 5 independent experiments and were analyzed with the unpaired two-tailed Student's t test. (B) Oxygen consumption rate (OCR) for TSH-N5 cells cultured in ULGM or ULGM supplemented with 4 mM lactate (ULGM+L) and the indicated concentrations of α-CHCA. Data are means ± SD from $n > 5$ biological replicates in one representative experiment ($N = 3$ experiments performed) and were analyzed by one-way ANOVA followed by Tukey's post hoc test. *$p < 0.05$, **$p < 0.01$, ****$p < 0.0001$.

Supplementary Materials and Methods

4.14. Sphere formation assay

Cells were plated in ultralow attachment 6-well plates (Corning, Corning, NY, USA) at a density of 2 × 10^4 cells per well and cultured for 5 days in ULGM or ULGM supplemented with 4 mM lactate. Images of 2 random fields per well, 3 wells per condition, were acquired with a BZ-X710 inverted microscope (Keyence, Osaka, Japan). Sphere number and size were quantified with the Keyence Analysis Software (Keyence, Osaka, Japan) and the number of spheres with a diameter >100 µm was counted.

4.15. Measurement of OCR

In preliminary experiments we determined the concentrations at which lactate transport inhibitor α-cyano-4-hydroxycinnamic acid (α-CHCA, Sigma) does not induce cell death but decreases lactate uptake in the tested cells. Oxygen consumption rate (OCR) was measured with a Seahorse XFe Extracellular Flux Analyzer (Agilent Technologies, Santa Clara, CA, USA). Cells were plated in 96-well plates (Agilent Technologies, Santa Clara, CA, USA) at 2 × 10^4 cells/well and incubated in NSM for 24 h. ULGM and ULGM+L (ULGM with 4 mM lactate) equivalents were prepared from glutamine- and pyruvate-free Seahorse XF DMEM (Agilent Technologies, Santa Clara, CA, USA) by supplementation with 0.175 mM glucose with or without 4 mM lactate. Cells were washed multiple times with ULGM, ULGM+L or ULGM+L and 2.5 mM or 5 mM α-CHCA and then incubated in the respective conditions for one hour before the start of the assay. OCR was measured repeatedly for 20 min and the basal OCR recorded 20 min after the initial measurement was compared between groups. Wells with more than 20% of cells detached at the end of the assay were excluded from analysis.
